# IFN-α as a time-sensitive biomarker during Oropouche virus infection in early and late seroconverters

**DOI:** 10.1038/s41598-019-54223-w

**Published:** 2019-11-29

**Authors:** Euzébio de Oliveira, Raimunda do Socorro Silva Azevedo, Jordana Grazziela Coelho-dos-Reis, Lis Ribeiro do Valle Antonelli, Milene Silveira Ferreira, Ana Carolina Campi-Azevedo, Matheus Fernandes Costa-Silva, Lívia Carício Martins, Jannifer Oliveira Chiang, Andréa Teixeira-Carvalho, Olindo Assis Martins-Filho, Pedro Fernando Costa Vasconcelos

**Affiliations:** 1 Instituto Evandro Chagas, Ananindeua, PA Brazil; 2 Universidade Federal de Minas Gerais, Belo Horizonte, MG Brazil; 3 Instituto René Rachou, Fundação Oswaldo Cruz – FIOCRUZ-Minas, Belo Horizonte, MG Brazil

**Keywords:** Viral infection, Prognostic markers

## Abstract

In the present study, patients with acute OROV fever were classified as early seroconverters (IgM/IgG positive at baseline) or late seroconverters (IgM/IgG negative at baseline) and the timeline kinetics of the production of chemokines and cytokines were assessed at 1–3, 4–7, 8–10 and ≥11 days after patients have reported the first symptoms. Regardless immunoglobulin profile, all OROV fever patients presented higher levels of CXCL8, and IFN-α and lower levels of TNF and IL-10 at baseline as compared to healthy donors (HD). Lower levels of CCL2, CXCL10, and IFN-γ and higher levels of CCL2, CXCL10, IL-6, and IL-17A were detected in early and late seroconverters, respectively, as compared to HD. While early seroconverters presented the increasing levels of CCL2 along the timeline, late seroconverters displayed decreasing levels of CCL2, CXCL10, and IL-6 following days of disease onset. Noteworthy was that IFN-α was revealed as universal biomarker of human OROV fever, while CXCL8 & IL-5 and CXCL10 & IL-17 were consistently observed in early and late seroconverters, respectively. Thus, our results suggest that the production of IFN-α, CXCL10, and IL-17 precede the seroconversion bringing novel insights on the immunological events triggered by the OROV disease.

## Introduction

*Oropouche virus* (OROV) belongs to one of the largest and most diversified families of RNA virus, the *Orthobunyaviridae*, which include several Arboviruses responsible for many important acute but self-limiting febrile illnesses in humans and also hemorrhagic fevers and encephalitis^1,2^. In regard to the epidemiologic aspects, a recent review reported that over the past 60 years, more than 30 outbreaks, comprising more than half a million clinical cases, have been attributed to OROV fever including affected areas in Brazil, Peru, Panama, Trinidad and Tobago. In Brazil, the Amazon region has been stricken for the largest OROV fever epidemics and is the largest endemic area for OROV^3,4^. In fact, until recently, OROV fever was the second most frequent arboviral infection in Brazil, surpassed only by dengue. Now due the recent *Chikungunya and Zika viruses* epidemics, OROV fever is considered the 4^th^ most prevalent arbovirus disease in the country. OROV is raising public agencies concern due to its prospective to spread geographically and emerge in naïve areas, which calls to its importance at an international panorama. The OROV disease starts by an infected arthropod bite, frequently by the midge *Culicoides paraensis*, its main urban vector, allowing for viral entry and replication in the muscle cells, followed by systemic dissemination to several organs and blood that results in high viremia detected between 2–4 days of symptoms onset^4^. Clinically, OROV fever can be characterized as acute febrile disease, with common symptoms and signs such as skin rash, headache, fever, muscle and joint pain^3^. The virus may cause meningitis, resulting in typical clinical manifestations of meningeal irritation; then it crosses the blood-brain barrier and infects neurons, producing encephalitis in more susceptible individuals^5^. Nevertheless, OROV fever is usually not diagnosed because of its mild and self-limited manifestations or eventually mistakenly diagnosed due to its similar clinical features with dengue, chikungunya, zika, other arboviral diseases and even malaria^4^.

Usually, serologic tests, using distinct antigens have been employed for the diagnosis of OROV infection based on the detection of specific IgG and IgM antibodies^4^. Despite the fact that seroconversion, detected by IgG and/or IgM ELISA upon OROV infection, is the “gold standard” method in epidemiological survey, in our clinical experience a time-dependent divergence in seroconversion after disease onset has been frequently observed. Indeed, while some patients already present IgM/IgG seropositivity upon disease onset, others exhibit late seroconversion profile, which may yield false negative diagnosis of OROV infection during the acute-phase disease. Little is known on the immunological events triggered during the infection that lead to early and late seroconversion after OROV fever. The analysis of immunological biomarkers may contribute to understanding of the mechanisms underlying this phenomenon.

In general, viral infections trigger the immune system resulting in activation of innate and adaptive cells toward the production of a strong inflammatory response mediated by several inflammatory cytokines and chemokines, such as type I interferon (IFN), that are essential for antiviral defense. In general, type I IFN signaling is well known to be able to trigger expression of several hundred of genes, involved in the blockage of viral entry, replication, translation and assembly, as well as modulate inflammation and adaptive immunity^6^. Type I IFN response is triggered at early stages of virus replicative cycle and its importance has being demonstrated in the mouse model of OROV disease^7^. Moreover, *in vitro* experiments have shown that IFN-β mRNA levels increase in the first hour post-infection and drop rapidly reaching very low levels at 24 hours post infection^7^. However, how cytokine kinetics develops during human OROV fever has not yet been reported.

Thus, the present study aimed at investigating soluble immunological biomarkers including circulating cytokines and chemokines in early and late seroconverters along days after OROV fever onset. The results show conspicuous differences in the cytokine and chemokine levels between these two groups, which suggest that the innate immune response pattern harnessed by OROV is closely associated to seroconversion.

## Results

### Serological status of patients with acute-phase OROV fever according to disease onset

**Figure 1:**
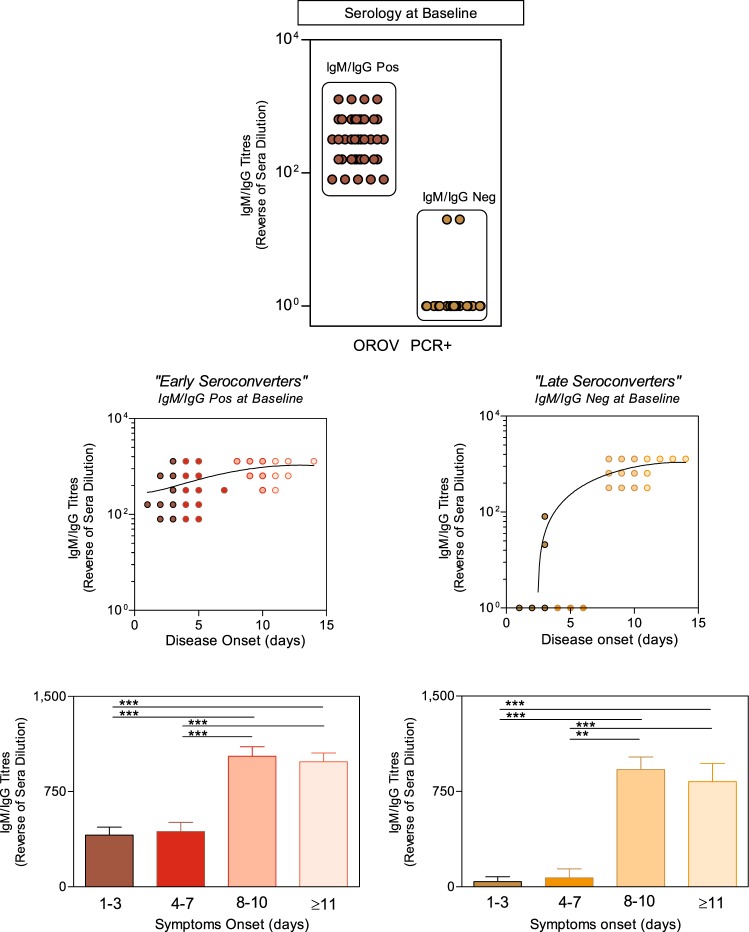
Serological profile of circulating IgM/IgG in acute-phase OROV fever patients at different days upon disease onset. IgM/IgG titers were measured by MAC-ELISA and OROV patients classified as positive (n = 48) or negative (n = 27), according to the IgM/IgG serology at baseline (top graph). Antibody titers were determined along the days after disease onset and displayed in scatter plot as the continuous-time to depict two clusters of OROV fever patients classified as early and late seroconverters (middle graphs). Bars charts represent the mean titers with standard errors (reverse of serum dilution) of 4 subgroups of early and late seroconverters, according to days of disease onset (1–3, 4–7, 8–10 and ≥11). Multiple comparisons amongst groups were carried out by Kruskal-Wallis test followed by Dunn’s post-test for sequential pairwise comparisons. Asterisks underscore differences amongst subgroups. **p ≤ 0.01, ***p ≤ 0.001 (bottom graphs).

In order to identify the profile of circulating IgM/IgG anti-OROV in patients with acute infection, serum samples from patients at different days upon disease onset were evaluated by MAC ELISA. Patients who were diagnosed as positive for OROV disease by RT-qPCR were subdivided as positive (IgM/IgGPos, n = 48) or negative (IgM/IgGNeg, n = 27) according to serology at baseline (Fig. 1, top panel). The results demonstrated a profile characteristic of early and late seroconversion. Two clusters were observed when IgM/IgG titers were evaluated: (i) patients displaying high titers since the beginning of disease onset, classified as early seroconverters and (ii) patients showing high titers around 8 days after disease onset, considered as late seroconverters (Fig. 1, middle panels). These two clusters were again subdivided into 4 groups according to days of disease onset: 1–3, 4–7, 8–10 and ≥11. IgM/IgG titers in early seroconverters were higher than late seroconverters at 1–3 and 4–7 subgroups.

However, both seroconversion clusters (early and late) presented distinct and significantly different IgM/IgG titers amongst early (1–3 and 4–7) as compared to late (8–10 and ≥11) time points (Fig. 1, bottom panels).

### Overall profile of circulating chemokines and cytokines of patients with OROV fever

**Figure 2:**
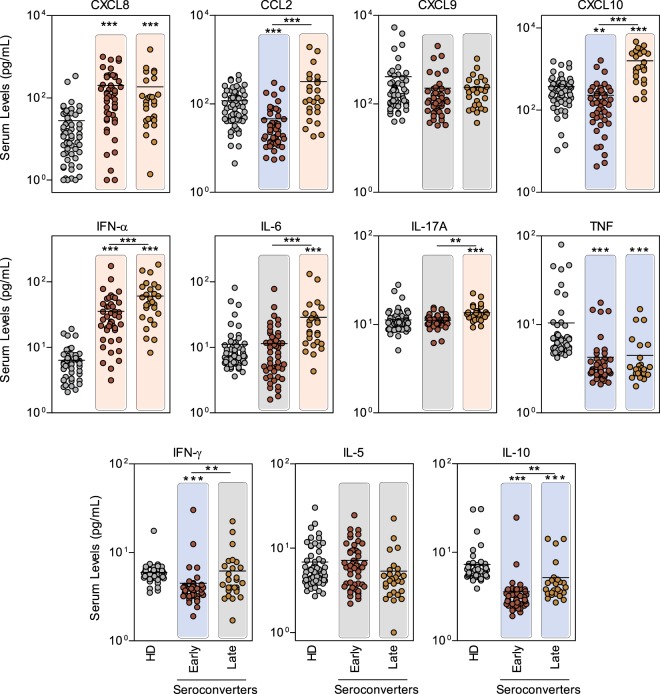
Levels of serum chemokines and cytokines in acute-phase OROV fever- patients with early and late seroconverters. Serum biomarkers (CXCL8, CCL21, CXCL9, CXCL10, IFN-α, IL-6, IL-17, TNF, IFN-γ, IL-5 and IL-10) were measured by CBA assay, as described in methods, in acute-phase OROV fever patients classified as early (n = 48, read circles) or late (n = 27, orange circles) seroconverters during disease onset and healthy donors (HD = 60, grey circles). Data are expressed as scattering of individual values (pg/mL). Multiple comparisons amongst groups were carried out by Kruskal-Wallis test followed by Dunn’s post-test for sequential pairwise comparisons and significant differences depicted as asterisks and connecting lines. Colored backgrounds highlighted increased (light pink), decreased (light blue) and unaltered (gray) levels of serum biomarkers in OROV subgroups as compared to HD, and differences are underscored by asterisks. *p ≤ 0.05, **p ≤ 0.01, ***p ≤ 0.001.

The overall chemokine and cytokine profiles were assessed by flow cytometry in plasma samples from acute-phase OROV fever patients categorized according to IgM/IgG serology results at baseline as early (IgM/IgGPos at baseline) or late (IgM/IgGNeg at baseline) seroconverters (Fig. 2). The results showed that OROV fever patients presented higher levels of CXCL8 and IFN-α (pink background) and lower levels of TNF and IL-10 than HD (blue background), regardless of antibody profile at baseline. Early seroconverters showed lower levels of CCL2, CXCL10 and IFN-γ (Fig. 2, blue background). Conversely, late seroconverters presented higher levels of CCL2, CXCL10, IL-6 and IL-17A as compared to HD (Fig. 2, pink background). Moreover, higher levels of CCL2, CXCL10, IFN-α, IL-6 and IL-17 were observed in late seroconverters as compared to early seroconverters (Fig. 2, pink background).

### Kinetics of circulating chemokines and cytokines of patients with acute-phase OROV fever

**Figure 3:**
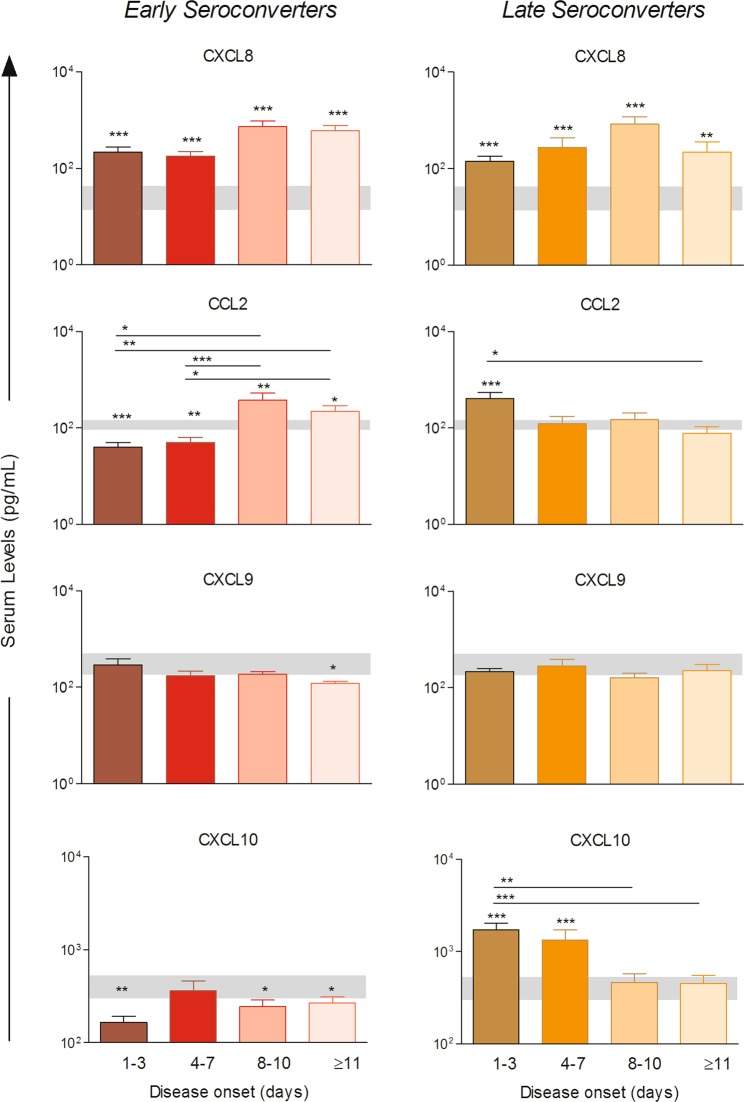
Kinetics of circulating chemokines in acute-phase OROV fever patients in different days upon disease onset. Levels of CXCL8, CCL2, CXCL9 and CXCL10 assessed 1–3, 4–7, 8–10 and ≥11 days upon disease onset in plasma of OROV fever patients considered as early seroconverters (n = 48, left panels) or late seroconverters (n = 27, right panel). Bars represent mean with standard errors (pg/mL) and grey boxes the reference range corresponding the 95%CI of the mean for the HD group. Multiple comparisons amongst groups were carried out by Kruskal-Wallis test followed by Dunn’s post-test for sequential pairwise comparisons and significant differences depicted as asterisks and connecting lines. Differences between HD and OROV fever subgroups in different days of disease are shown as asterisks. *p ≤ 0.05, **p ≤ 0.01, ***p ≤ 0.001.

**Figure 4:**
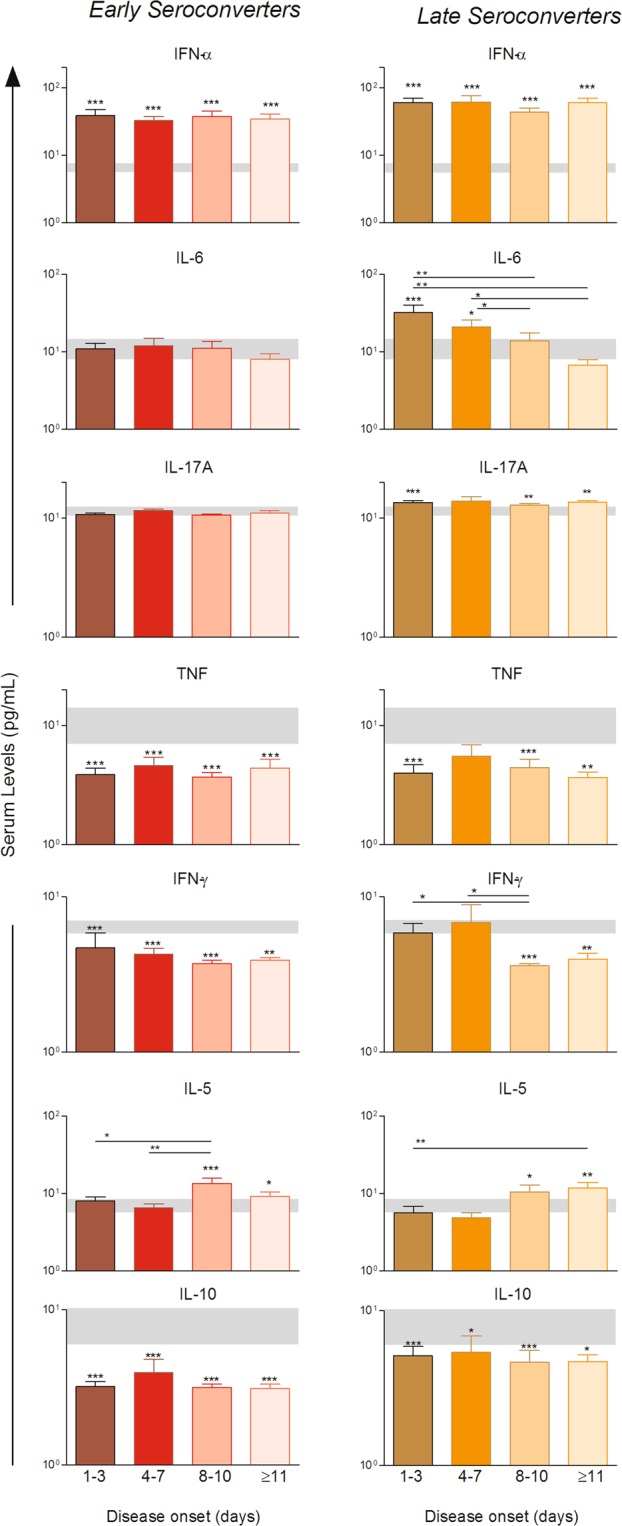
Kinetics of circulating cytokines in acute-phase OROV fever patients in different days upon disease onset. Levels of IFN-α, IL-6, IL-17, TNF, IFN-γ, IL-5 and IL-10 assessed 1–3, 4–7, 8–10 and ≥11 days upon disease onset in plasma of OROV fever patients considered as early seroconverters (n = 48, left panels) or late seroconverters (n = 27, right panel). Bars represent mean with standard errors (pg/mL) and grey boxes the reference range corresponding the 95%CI of the mean for the HD group. Multiple comparisons amongst groups were carried out by Kruskal-Wallis test followed by Dunn’s post-test for sequential pairwise comparisons and significant differences depicted as asterisks and connecting lines. Differences between HD and OROV fever subgroups in different days of disease are shown as asterisks. *p ≤ 0.05, **p ≤ 0.01, ***p ≤ 0.001.

The timeline kinetics of chemokine and cytokine levels were determined at 1–3, 4–7, 8–10 and ≥ 11 days after disease onset in plasma of OROV fever patients classified as early and late seroconverters (Figs. 3 and 4). Higher levels of CXCL8 were observed throughout the timeline kinetics in both groups as compared to HD (grey zone). Distinct kinetics of CCL2 production was observed in early versus late seroconverters. While early seroconverters displayed lower levels of CCL2 at 1–3 and 4–7 days and higher levels at 8–10 and ≥ 11 days after disease onset, late seroconverters displayed higher levels only at 1–3 days of disease onset as compared to both HD and the time point ≥ 11 days after disease onset. Lower levels of CXCL9 were observed in early seroconverters at ≥ 11 days after disease onset. Interestingly, lower levels of CXCL10 were observed in almost all the time points tested in patients classified as early seroconverters, while in those considered late seroconverters the CXCL10 production was augmented at 1–3 and 4–7 days of disease onset as compared to HD. Moreover, in late seroconverters, higher levels of CXCL10 were also observed at 1–3 days as compared to 8–10 and ≥ 11 days after disease onset (Fig. 3).

In regard to cytokine production, the levels of IFN-α were higher throughout the timeline kinetics in both groups of patients when compared to HD (grey zone). In general, the production of TNF, IFN-γ and IL-10 were lower in both groups of patients as compared to HD. The levels of IL-5 were significantly increased in patients at 8–10 and ≥ 11 days after disease onset as compared to HD. On the contrary, higher levels of IL-6 and IL-17A were observed at 1–3 and 4–7 days after disease onset in late seroconverters in comparison to HD (Fig. 4).

### Plasmatic biomarker signatures in acute-phase OROV fever

**Figure 5:**
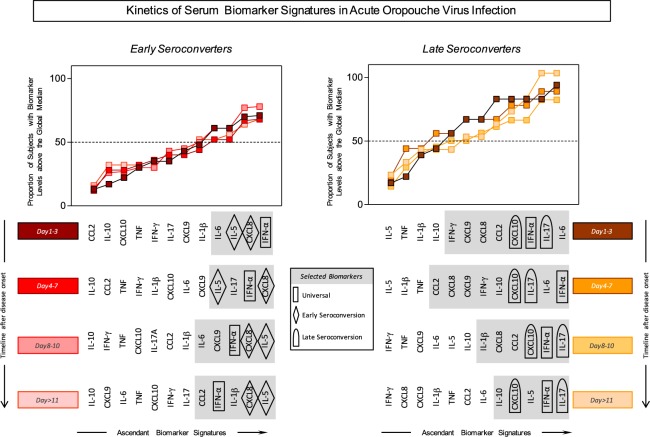
Kinetics of serum biomarker signature in acute-phase OROV fever. Signature curves were plotted using the global median of each cytokine (pg/mL) index as the cut-off mark to distinguish high and low producers of each cytokine/chemokine from early (left panel) and late seroconverters (right panel). Each line corresponds to the biomarker signatures at different days after disease onset to identify changes in the overall cytokine/chemokine profile along time of infection. Grey boxes highlight those biomarkers with levels above the global median observed in more than 50% of OROV fever patients. Geometric symbols (rectangle, diamond and bullet) depict those biomarkers considered “universal” and those exclusively found in “early seroconverters” and “late seroconverters”, respectively.

In order to evaluate the overall profile of soluble putative biomarkers in early and late seroconverters upon different days of disease onset, the continuous data was converted into categorical data. For that, the global median of all groups tested was calculated and applied as the cut-off point to distinguish between high and low producers of each chemokine and cytokine. This strategy allowed for generating the proportion of subjects with biomarker levels above the global median, which were assigned as high producers. The proportion of high producers was plotted in an increasing fashion, creating the ascendant signature for each and every biomarker (left to right) at each time point tested (top to bottom). The biomarkers with proportion of high producers above the 50^th^ percentile were selected in grey. Among these, three patterns of altered biomarkers were observed and highlighted with symbols as depicted in Fig. 5.

In general, a broader spectrum of biomarker with augmented proportion of high producers was observed in late seroconverters at 1–3 and 4–7 days after disease onset as compared to those early seroconverters.

Noteworthy was that IFN-α signature was consistently high in all time points tested in both early and late seroconverters and considered as the universal biomarker of human acute-phase OROV fever. The proportion of CXCL8 and IL-5 high producers were persistently elevated throughout the timeline kinetics selectively amongst early seroconverters. Conversely, the proportion of CXCL10 and IL-17A high producers were highlighted throughout all time points tested for late seroconverters.

## Discussion

Oropouche virus (OROV) stands out as the utmost prevalent *Orthobunyavirus* contagion in some northern South American countries and specially in the Amazon region. In these areas, OROV is endemic, nonetheless scarce information exists about the inflammatory response induced by OROV fever. The present study is the first comprehensive investigation on the chemokine and cytokine profile in early and late seroconverters with OROV fever. In regard to the initial responses triggered by OROV, the innate antiviral response induced by OROV is mediated by induction of type I IFN pathway through Interferon Regulatory Transcription Factors (IRF3 and IRF7), as well as interferon-α/β receptor, and Mitochondrial Antiviral Signaling Protein (MAVS), as evidenced in animal model-based studies^7^. The OROV fever pathogenesis was in fact described as restricted by the IFN pathway-related signaling molecules MAVS, IRF-3, IRF-7 in non-myeloid cells^7^. In line with these findings, a very elegant study demostrated that several *Orthobunyaviruses*, including: *Tacaiuma (TCMV), Guama (GMAV), Guaroa (GROV) and Caraparu (CARV)* viruses in addition to OROV are vulnerable to the antiviral attack mediated by Interferon-α in time and dose-dependent fashion^8^. In fact, OROV fever is restrained by the IFN pathway in wild-type mice. This is exemplified by the fact that IFN-α knockout mice display increased hepatic injury, significantly superior mortality and rapid progress of the disease^7^, as similar as previously observed into immunocompetent Syrian golden hamsters^9^. It is important to underscore that although IFN-α is a useful universal biomarker for OROV fever, it should be considered together with other clinical and laboratorial records since several other viruses can also elicit IFN-α production.

In addition to IFN-induced responses, TNF signaling components such as TNF-Receptor Associated Factor 3 (TRAF3) and Stimulator of Interferon Genes (STING) seem to be important for OROV innate immunity-mediated control. Conversely, as the infection progresses, these mediators are down-modulated in a virus-dependent manner. Possibly, the virus tries to escape the antiviral cellular pathways by increasing the expression of short non-coding cognate miRNAs^10^. Thus, the only cytokine ever assessed during OROV fever was type I IFN and it has been demonstrated to be essential for resistance in mouse model.

In agreement with these findings, OROV patients presented higher levels of IFN-α indicating strong activation and lower levels of TNF and IL-10 than those by HD, regardless of antibody profile at baseline. IFN-α was the sole cytokine presented in higher levels in both early and late converter patients, as evidenced by the biomarker signatures. In fact, IFN-α was the only mediator present in all time-points upon disease onset and in both early and late seroconverters, indicating possibly that the type I IFN-mediated antiviral responses may not be directly connected to antibody production.

In the present study, we demonstrate that OROV fever patients with early and late seroconversion profile display divergent pattern of inflammatory response as assessed by measuring circulating chemokines and cytokines. The early seroconverters display high levels of CXCL8 and IL-5 in all time-points. The high production of IL-5 observed in early seroconverters is consistent with the role of this cytokine in inducing B cell activation and differentiation into antibody producing-plasma cells. This finding may suggest that these patients are ahead in the timeline of infection or this may reflect an intrinsic ability of certain hosts to mount a more robust immune response to OROV. Another possibility is that these early seroconverters may display a distinct serological pattern due to cross reactivity generated by previous infection with other *Orthobunyavirus*. The later hypothesis is less likely probable, considering that during serological screening at baseline seronegativity was observed for *Caraparu* and *Catu viruses* (titers <1:40) for these patient subgroup and during follow up developed a strong seropositivity to OROV (titers from 1:640 to 1:1,280).

High producers of CXCL10 and IL-17 were a hallmark of OROV fever late seroconverters. However, as the levels of IL-17 were particularly low in all OROV patients tested, it is a challenge to validate this molecule as a biomarker for laboratorial follow up of OROV fever. On the other hand, substantial levels of CXCL10 were observed in late but not in early soroconverters. High levels of CXCL10 have been already reported in other virus infection^11,12^. Similar pattern of production was observed for CCL2, a chemokine associated with monocyte recruitment, essential for viral clearance^12^. In fact, CXCL10 and CCL2 are both closely associated with innate immune events that could support type I IFN responses. Taking into account these results herein obtained, it is clear that CXCL10 and CCL2 are also closely associated with immune response after a natural acquired *Orthobunyavirus* disease and could represent complementary biomarkers for OROV fever follow up in clinical practice.

The reasons for the different seroconversion profiles may be attributed to idiosyncratic events intrinsic to the divergent and miscegenated genetic background of the Brazilian population residing in the Amazon region, as well as to the abundant and diversified plethora of arboviruses, including different orthobunyaviruses with capacity to infect and cause disease in humans to which this population is under risk of exposition. It is plausible to hypothesize that preceding contact to different orthobunyavirus antigens may influence the immune response at least to acute-phase OROV fever. In fact, patients included in this study reside in an endemic area to several *Flaviviruses* such as *Dengue virus*, *Yellow fever* *virus* and *Zika virus*, and potentially also to different *Orthobunyaviruses* such as groups C, *Guamá, Anopheles A, California*, and others, which have caused several seasonal and non-seasonal outbreaks with co-circulation of different arboviruses^13^. Viremia is an interesting biomarker that would contribute to understand the different seroconversion profiles. However, no clear association was found amongst the viremia level and neither the chemokine/cytokine profile, nor the seroconversion status observed for the samples included in the present investigation (data not shown).

Aiming at determining whether the distinct seroconversion profile as well as chemokine/cytokine patterns were somehow associated with symptoms, we have performed several data mining approaches, but no association could be identified between single or combined clinical records and serum biomarkers. In fact, during OROV disease, the mechanisms underlying protection and pathogenesis have not been yet clearly defined. All in all, the present study has identified IFN-α as a universal biomarker of human OROV fever, while CXCL8 & IL-5 and CXCL10 & IL-17 were observed consistently in early and late seroconverters, respectively. Thus, our results suggest that production of IFN-α and CXCL10 precede the seroconversion bringing novel insights on disease pathogenesis i.e. the immunological events triggered by the OROV fever.

## Material and Methods

### Study population

The present study is a cross-sectional observational study that includes a total of 135 serum samples obtained from the blood bank at the Foundation Center of Hemotherapy and Hematology of Pará (HEMOPA) and Evandro Chagas Institute (IEC). Sixty healthy donors (HD - HEMOPA) and 75 Oropouche-exposed patients (OROV - IEC) were enrolled. HD specimens were triaged for several blood-borne pathogens and classified as fully suitable for donation of blood. OROV fever group was composed of patients clinically investigated by researchers of the Evandro Chagas Institute during the OROV fever outbreaks that occurred in 2006 at Magalhães Barata (Latitude: 0° 47′ 53″ S, Longitude: 47° 36′ 10″ W) in Pará State, and in 2009 at Mazagão (Latitude: 0° 6′ 58″ S, Longitude: 51° 17′ 10″ W) in Amapá State Brazil. OROV fever patients were enrolled upon disease onset, and symptoms were assessed according to Vasconcelos *et al*., 2009: fever (100%), headache (61%), dizziness (54%), myalgia (48%), photophobia (39%), nausea (34%), vomiting (33%), arthritis (18%), epigastric pain (8%), and exanthema (4%). Diarrhea and conjunctival congestion were not observed. All subjects included in this study signed a written informed consent. The samples stored at −80 °C were used upon signed agreement (080/2011 – GAPRE/HEMOPA). The study protocol was approved by the Ethics Committee at Instituto Evandro Chagas (Plataforma Brasil, CAAE#0008.0.72.000-10) and all methods were performed in accordance with the relevant guidelines and regulations.

### Molecular and serological IgM and IgG tests for Oropouche virus infection diagnosis

Sera were tested for the presence of OROV with established reference assays based on real-time reverse transcription polymerase chain reaction (RT-qPCR). RT-qPCR showed high titer of OROV in acute-phase serum samples from febrile patients. The detection of anti-OROV IgM and IgG antibodies was performed by MAC ELISA and hemagglutination-inhibition (HI) as previously described^3,14^. All samples were also screened for Caraparu and Catu infection and presented negative results at baseline (titer < 1:40).

### Cytokine and Chemokine assessment

The plasma levels of chemokines (CXCL8, CCL2, CXCL9, CXCL10) and cytokines (IFN-α, IL-6, IL-17A, TNF, IFN-γ, IL-5, and IL-10), of the sera from the patients selected for this research, were quantified using the Cytometric Bead Array (BD, Pharmingen, USA), which employs a mixture of polystyrene beads of discrete and distinct fluorescence intensities and coated with antibodies specific for human chemokines and cytokines. This methodology allows the simultaneous evaluation of several soluble biomarkers in the same assay using small sample volumes. Plasma and human cytokine standards were assayed according to the manufacturer’s instructions and immediately acquired on a FACScalibur^TM^ flow cytometer (BD, San Jose, CA, USA). The FCAP Array software, v1.0 (San Jose, CA, USA) was used for data analysis. An adjustment model through the curve of the 4^th^ logistic parameter, which allows the adjustment of the best non-linear curve for detectable data, was employed and the results were expressed in pg/mL.

### Statistical analysis

Conventional statistical analysis was performed using GraphPad Prism Software v.8.0 (San Diego, CA, USA). Multiple comparisons amongst groups were carried out by Kruskal-Wallis test followed by Dunn’s post-test for sequential pairwise comparisons. In all cases, the differences were considered significant if p < 0.001 (***), p < 0.01 (**) and p < 0.05 (*). The biomarker signature analysis was plotted as described previously by Luiza-Silva *et al*.^15^. Briefly, the global median values of cytokine and chemokine plasma concentrations were calculated and employed as the threshold to identify the proportion of subjects as low (below global median) or high producers (above global median) of each biomarker. The cutoff of 50% was considered to define biomarkers, which are universal, present during early and late seroconversion.

## Data Availability

The datasets generated during and/or analyzed during the current study are available from the corresponding author on reasonable request.
